# A Case of Leptospirosis-Associated Severe Pulmonary Hemorrhagic Syndrome Successfully Treated with Venovenous Extracorporeal Membrane Oxygenation

**DOI:** 10.1155/2017/5369267

**Published:** 2017-10-25

**Authors:** Nao Umei, Shingo Ichiba

**Affiliations:** Department of Surgical Intensive Care Medicine, Nippon Medical School Hospital, Tokyo, Japan

## Abstract

**Background:**

In patients with leptospirosis-associated severe pulmonary hemorrhagic syndrome (SPHS), hypoxemia is the most common cause of death despite maximal mechanical ventilation.

**Case:**

A 50-year-old male sushi chef who had never traveled outside Japan presented with a 2-day history of fever and muscle pain. On admission, the patient had thrombocytopenia, renal insufficiency, and jaundice. His condition continued to deteriorate, with decreasing platelet count, worsening renal function, hyperbilirubinemia, hypotension, and respiratory distress. On day 5 after onset of symptoms, he required intubation and mechanical ventilation. Bronchoscopy showed diffuse endobronchial bleeding. His respiratory status worsened rapidly with a partial pressure of arterial oxygen to fraction of inspired oxygen ratio of 70, necessitating venovenous extracorporeal membrane oxygenation (V-V ECMO) and treatment with an inotrope, renal replacement therapy, and broad-spectrum antibiotics including benzylpenicillin. Anticoagulation was maintained at the minimum level. His condition improved, and he was weaned off ECMO on day 15 and discharged on day 19 after onset of symptoms. The leptospirosis diagnosis was confirmed by leptospiral DNA detection in urine samples by polymerase chain reaction and the results of paired serum antibody titer testing.

**Conclusions:**

V-V ECMO may prevent mortality in patients with leptospirosis-induced SPHS that does not respond to conventional therapy.

## 1. Introduction

Leptospirosis presents with acute influenza-like symptoms such as headache, chills, fever, vomiting, and muscle pain. Although leptospirosis can present with mild nonspecific flu-like symptoms, in its severe form, called Weil disease, it causes symptoms such as jaundice, bleeding, and renal failure [1, 2]. Rapid hemorrhagic tendency, jaundice, renal dysfunction, rhabdomyolysis, and myocardial pericarditis appear 4-5 days after disease onset. Jaundice, bleeding, and impaired renal function usually diminish after several weeks. However, pulmonary involvement occurs in 20–70% of leptospirosis cases, with pulmonary hemorrhage constituting a major cause of death [3–6]. The mortality rate in leptospirosis cases with severe pulmonary hemorrhagic syndrome (SPHS) is 30–60% even with adequate treatment [4, 7]. In this report, we describe a case of life-threatening leptospirosis with SPHS that occurred in urban Tokyo and that was successfully managed with venovenous extracorporeal membrane oxygenation (V-V ECMO).

## 2. Case Presentation

A 50-year-old male sushi chef who had never traveled outside Japan presented with a 2-day history of fever and muscle pain. Laboratory investigation yielded the following results: white blood cells (WBC): 7780/*µ*L; platelets: 89000/*µ*L; C-reactive protein (CRP): 36.4 mg/dL; blood urea nitrogen (BUN): 47 mg/dL; creatinine: 2.41 mg/dL; and total bilirubin (T-Bil): 2.0 mg/dL. The day after he was admitted to the hospital, the patient's condition worsened, as indicated by the laboratory data: WBC: 6700/*μ*L; platelets: 50000/*µ*L; CRP: 32.49 mg/dL; BUN: 66 mg/dL; creatinine: 2.81 mg/dL; and T-Bil: 5.6 mg/dL. He was presumptively diagnosed with bacterial pneumonia, atypical pneumonia, and miliary tuberculosis. He was also diagnosed with leptospirosis on the basis of jaundice, renal failure, and thrombocytopenia. He was administered the broad-spectrum antibiotics ceftriaxone, levofloxacin, and minocycline on the first day of admission to the referral hospital. Vancomycin and meropenem were administered on the second day. He was also given the antitubercular agents—isoniazid, rifampicin, pyrazinamide, and ethambutol—even though the results of a tuberculosis screening test were negative. Despite these treatments, his systolic blood pressure dropped to 70 mmHg, and his respiratory condition deteriorated; he was intubated 5 days after the onset of symptoms, which is three days after admission to the previous hospital. An X-ray showed bilateral alveolar infiltrates, and blood gas analysis revealed the following: pH: 7.390; partial pressure of arterial carbon dioxide (PaCO_2_): 28.3 mmHg; and partial pressure of arterial oxygen (PaO_2_): 70.4 mmHg HCO_3_^−^ 16.8 mmol/L with fraction of inspired oxygen (*F*_I_O_2_) of 1.0 as well as positive end-expiratory pressure (PEEP) of 10 cm H_2_O. The patient developed a life-threatening condition with septic shock and severe acute respiratory failure, despite optimal medical treatment. He was referred to our center for ECMO. Our ECMO team decided to initiate V-V ECMO at the previous hospital and transport the patient on V-V ECMO because the patient was severely hypoxic and needed maximal doses of norepinephrine, epinephrine, and vasopressin to stabilize his cardiac condition. Cannulation was performed via the right femoral vein with 25-French drainage cannulae (HLS Cannulae Maquet Cardiopulmonary, Hirrlingen, Germany) for access and via the right internal jugular vein with 23-French cannulae for return. The ECMO circuit was an adult ECMO bypass custom tubing pack consisting of a Rotaflow® centrifugal pump (Maquet Cardiopulmonary GmbH, Hirrlingen, Germany) and gas exchanger (MERA NHP Excelung NSH-R HPO-23WH-C®, Senko Medical Inc., Tokyo, Japan). V-V ECMO was initiated at a blood flow rate of 4.0 L/min, with sweep gas flow through the oxygenator at 4.0 L/min of 100% oxygen. After the procedure, the patient's SpO_2_ was 94%, heart rate was 131 beats/min, and blood pressure was 142/74 mmHg on 0.1 *μ*g/kg/min of epinephrine and 0.3 *μ*g/kg/min of norepinephrine. He was transported to our center via ground ambulance.

After he was transported to our ICU, we maintained the ECMO blood flow at >3.0 L/min during the first 3 days of ECMO because his activated partial thromboplastin time was maintained at around 40–50 s, which was lower than normal because of bleeding (Table 1). After bleeding was controlled, we decreased the ECMO blood flow gradually to 1.5 L/min when peripheral capillary oxygen saturation (SpO_2_) was over 90%. During V-V ECMO the mechanical ventilation was set at the lung rest setting, which consisted of a driving pressure of 5 cm of water, PEEP of 12 cm of water, and *F*_I_O_2_ of 0.4. Fiber-optic bronchoscopy showed diffuse endobronchial bleeding. The patient was systemically heparinized to maintain an activated partial thromboplastin time of 40–50 seconds, which was lower than the normal value for V-V ECMO. Dobutamine was initiated at 3 *µ*g/kg/min because transthoracic echocardiography showed that the ejection fraction was 30%, mitral regurgitation was moderate, and tricuspid regurgitation was severe. After initiation of dobutamine, his cardiac function improved, epinephrine was stopped the next day, and norepinephrine was stopped the day after that. He remained oliguric and needed renal replacement therapy for 7 days. At the time of presentation to our center, his WBC count was 18,000/*µ*L, CRP level was 24.52 mg/dL, and procalcitonin level was > 100 ng/mL. Although we thought of a wide variety of infections as differential diagnoses, we strongly suspected leptospirosis on the basis of severe acute respiratory failure, myocardial pericarditis, renal failure, jaundice, and thrombocytopenia, which made us continue the broad-spectrum antibiotics, levofloxacin, vancomycin, meropenem, antitubercular agents, and add benzylpenicillin. Because of pulmonary hemorrhage, he was given a platelet transfusion (20 units) for the first 3 days of V-V ECMO therapy. Even though his pulmonary hemorrhage improved on day 8 of V-V ECMO, computed tomography scans showed bilateral ground-glass opacities, bilateral pleural effusion, and atelectasis. Rehabilitation, including sitting on the edge of the bed and standing, was initiated. On day 6 of ECMO therapy, polymerase chain reaction analysis of urine samples collected at the previous hospital confirmed the presence of leptospiral DNA. The tests for tuberculosis,* Legionella* urinary antigen, pneumococcal antigen, serum* Mycoplasma* antibody, and* Chlamydia pneumoniae* antibody all yielded negative results. We switched the intravenous antibiotics from broad-spectrum antibiotics to benzylpenicillin (12 million units/day).

**Figure 1:**
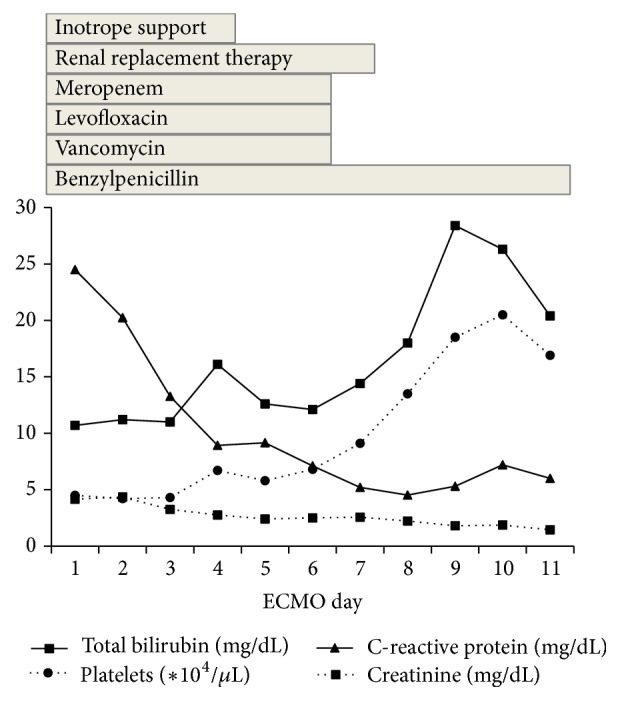
Summary of laboratory data and therapeutic course.

**Figure 2:**
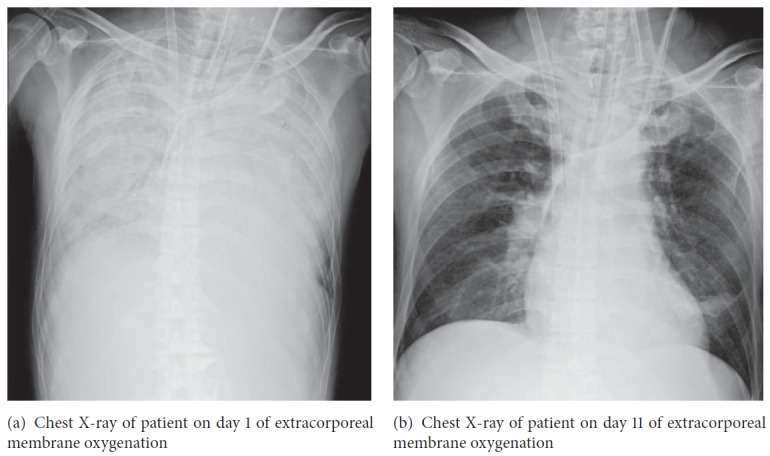


**Figure 3:**
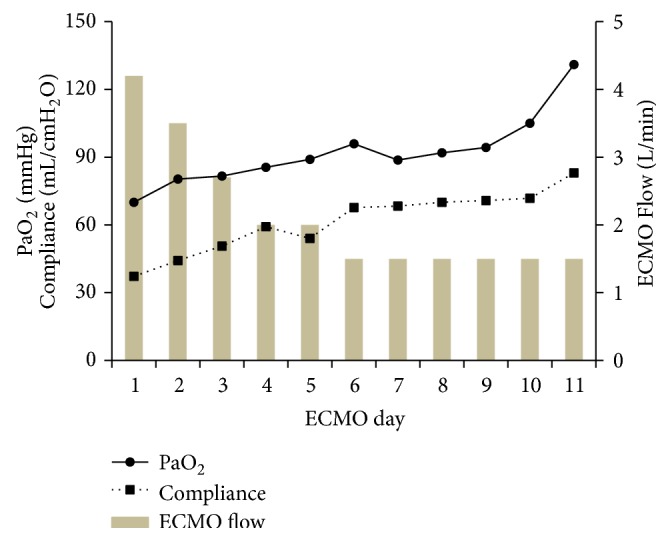
Partial pressure of arterial oxygen (PaO_2_) and compliance during extracorporeal membrane oxygenation therapy.

On day 11 of ECMO therapy, blood tests showed improvements in platelet count and serum CRP, creatinine, and T-Bil levels (Figure 1). In addition, the amount of sputum decreased, and improvements were observed in his chest X-ray (Figure 2), compliance, and oxygenation (Figure 3); therefore, we performed a 2-hour trial off-test. The patient tolerated the trial without O_2_ flow to the oxygenator; therefore, we decided to wean him off ECMO. He was extubated 2 days after being taken off ECMO and transferred back to the previous hospital 2 days after extubation. His last blood gas analysis revealed the following results: pH: 7.468; PaCO_2_: 36.6 mmHg; PaO_2_: 67.2 mmHg; and HCO_3_^−^: 26.2 mmol/L on room air. Laboratory investigation revealed the following results: WBC: 7400/*µ*L; platelets: 256000/*µ*L; CRP: 1.13 mg/dL; BUN: 29.1 mg/dL; creatinine: 1.36 mg/dL; and T-Bil: 7.9 mg/dL. Finally, paired serum antibody titer testing (on days 5 and 19 after the onset of symptoms) showed a fourfold or higher increase in antibody titers for* Leptospira interrogans* serovar Copenhageni and* Leptospira interrogans* serovar Icterohaemorrhagiae, confirming the diagnosis of leptospirosis.

## 3. Discussion

In the present report, we describe the case of a patient with leptospirosis-associated SPHS combined with jaundice, acute renal failure, and cardiomyopathy. The present case report is important because our findings suggest the possibility of improving patient survival by using V-V ECMO in patients with leptospirosis-associated SPHS.

In most leptospirosis cases, the pulmonary symptoms usually appear 4–6 days after disease onset. The disease may progress rapidly and result in death in less than 72 hours [8]. SPHS, also described as the severe pulmonary form of leptospirosis (SPFL), is believed to be the major form of death in leptospirosis infection [8]. SPFL also manifests as pulmonary hemorrhage, which is usually massive and leads to respiratory insufficiency and death by asphyxiation. It is also similar to the pathological expression of diffuse alveolar hemorrhage [9]. To our knowledge, there are no reports comparing leptospirosis-associated SPHS with nonleptospirosis SPHS. We believe that the fundamental strategy for the treatment of pulmonary insufficiency due to the clinicopathological features mentioned above would be identical for both conditions, including mechanical ventilation with higher PEEP and management of coagulopathy. In some catastrophic cases with severe hypoxemia unresponsive to conventional medical treatment, ECMO is required, under cautious anticoagulation therapy.

The pathophysiology of pulmonary injury in leptospirosis is poorly understood. The pulmonary injury may be caused by an undefined leptospiral toxin that induces endothelial damage in pulmonary capillaries, or by host immune responses [6, 10, 11]. Methylprednisolone has been used for treatment of SPHS on the basis of the pathogenetic mechanism of lung injury; however, no significant mortality benefit has been observed in patients already on mechanical ventilation [12]. Trivedi et al. evaluated the efficacy of cyclophosphamide and plasma exchange in patients with leptospiral pulmonary hemorrhage. However, patients with severe disease may not tolerate the transient hypoxemia associated with plasma exchange [13]. High-frequency oscillatory ventilation may be effective for pulmonary hemorrhage treatment; however, it may result in significant hemodynamic instability [14]. Alternatively, V-V ECMO, which supports pulmonary function and minimizes the damage induced by mechanical ventilation, could be useful until the lungs recover. Liao et al. have reported the successful use of V-V ECMO for the treatment of severe respiratory failure due to alveolar bleeding and acute respiratory distress syndrome [15]. They initiated ECMO on day 4 because the PaO_2_/*F*_I_O_2_ ratio was 162; ECMO was maintained for 6 days, and extubation was performed 4 days after ECMO discontinuation. Arokianathan et al. reported that, after transportation to the ECMO center, the patient was maintained on ECMO for 8 days and extubated 2 days after ECMO discontinuation [16]. In the present case, ECMO was initiated on day 5 after onset of symptoms, when SPHS developed, and continued for 11 days; the patient was extubated 2 days after ECMO discontinuation.

Liao et al. used 4 units of platelets, 12 units of red cell concentrate, and 24 units of fresh frozen plasma during ECMO management [15]. The transfusion of 60 units of platelets was required to maintain the platelet count at >50,000/*µ*L, which is the limit specified in our ECMO management protocol for patients with bleeding complications. We also administered 8 units of red cell concentrate and 4 units of fresh frozen plasma because of massive pulmonary alveolar bleeding. Although blood transfusions are inevitable for the management of alveolar bleeding, ECMO is useful as an adjunct therapy until alveolar bleeding stops and respiratory condition improves in patients with leptospirosis.

Our patient's WBC count was 7780/*μ*L on the day of his admission to the previous hospital, which was not very high. De Silva et al. reported in their large prospective study that total white cell counts showed a decline over the first 5 days of illness and then rose until the end of second week [17]. It is supposed that the delayed increase in the WBC count may have coincided with the initiation of lung injury, which was also seen in our patient's clinical course.

The cause of severe respiratory failure in the present case was massive alveolar bleeding and pulmonary edema. Marked alveolar bleeding was observed during bronchoscopy. In addition, cardiogenic pulmonary edema secondary to myocarditis may make respiratory failure worse. Moreover, the Jarisch-Herxheimer reaction may be associated with deterioration of respiratory status [18].

Leptospirosis is endemic in tropical regions and extremely rare in urban areas. However, patients without a history of traveling to tropical regions such as Southeast Asia and Latin America can develop leptospirosis in urban areas. If a patient has flu-like symptoms and multiple organ failure, it is important to rule out leptospirosis as a differential diagnosis. The course of the disease should be carefully monitored in the intensive care unit as respiratory distress may progress rapidly. In addition, V-V ECMO therapy should be considered early in case of difficulty in maintaining adequate oxygenation by conventional mechanical ventilation because respiratory distress is the most common cause of death in these cases.

In conclusion, we successfully treated a patient with leptospirosis-associated SPHS by using V-V ECMO with careful anticoagulation therapy. Our findings indicate that the survival of patients with leptospirosis could be improved by using V-V ECMO.

## Figures and Tables

**Table 1:** Extracorporeal membrane oxygenation settings.

ECMO day	1	2	3	4	5	6	7	8	9	10	11
Flow (L/min)	4.2	3.5	3	2	2	1.5	1.5	1.5	1.5	1.5	1.5
Gas flow (L/min)	4	4	4	4	4	4	4	4	4	4	4
*F*_D_O_2_	1	1	1	1	1	1	1	1	1	1	1
Motor RPM	2865	2500	2300	1800	1800	1500	1500	1500	1500	1500	1500

*F*_D_O_2_: fraction of delivered oxygen to the oxygenator; RPM: revolutions per minute.
